# Upregulated expression of transforming growth factor-β receptor I/II in an endemic Osteoarthropathy in China

**DOI:** 10.1186/s12891-021-04939-6

**Published:** 2021-12-20

**Authors:** Ying Zhang, Yudong Mu, Ying He, Zhengzheng Li, Ge Mi, Yinan Liu, Meng Zhang, Hui Wang, Yiping Feng, Qian Fang, Tianyou Ma, Xianghua Deng, Jinghong Chen

**Affiliations:** 1 The Institute of Endemic Disease, School of Public Health, Health Science Center of Xi’an Jiaotong University, Xi’an, Shaanxi 710061 P.R. China; 2 School of Nursing, Health Science Center, Xi’an Jiaotong University, Xi’an, Shaanxi 710061 P.R. China; 3 Department of Clinical Laboratory, Tumor Hospital of Shaanxi Province, Affiliated to the Medical Collage of Xi’an Jiaotong University, Xi’an, Shaanxi 710061 P.R. China; 4 Research Division, HSS, Research Institute, Hospital for Special Surgery, and Weill Cornell Medical College, 535 East 70th Street, New York, NY 10021 USA

**Keywords:** TGF-β receptor II, TGF-β receptor I, Se-deficiency, T-2 toxin, Kashin–Beck disease

## Abstract

**Background:**

Kashin–Beck disease (KBD) is a chronic, deforming, endemic osteochondropathy that begins in patients as young as 2–3 years of age. The pathogenesis of KBD remains unclear, although selenium (Se) deficiency and T-2 toxin food contamination are both linked to the disease. In the present study, we evaluated transforming growth factor-β receptor (TGF-βR I and II) levels in clinical samples of KBD and in pre-clinical disease models.

**Methods:**

Human specimens were obtained from the hand phalanges of eight donors with KBD and eight control donors. Animal models of the disease were established using Sprague–Dawley rats, which were fed an Se-deficient diet for 4 weeks and later administered the T-2 toxin. Cartilage cellularity and morphology were examined by hematoxylin and eosin staining. Expression and localization of TGF-βRI and II were evaluated using immunohistochemical staining and western blotting.

**Results:**

In the KBD samples, chondral necrosis was detected based on cartilage cell disappearance and alkalinity loss in the matrix ground substance. In the necrotic areas, TGF-βRI and II staining were strong. Positive percentages of TGF-βRI and II staining were higher in the cartilage samples of KBD donors than in those of control donors. TGF-βRI and II staining was also increased in cartilage samples from rats administered T-2 toxin or fed on Se-deficient plus T-2 toxin diets.

**Conclusion:**

TGF-βRI and II may be involved in the pathophysiology of KBD. This study provides new insights into the pathways that contribute to KBD development.

**Supplementary Information:**

The online version contains supplementary material available at 10.1186/s12891-021-04939-6.

## Background

Kashin–Beck disease (KBD) is a deforming endemic osteochondropathy. The disease can begin in patients aged 2 or 3 years and presents with osteoarthropathy between the ages of 5 and 13 years [1]. In 2016, 22,567,600 people were diagnosed with this disease, among whom 574,925 were diagnosed in China [2]. Symptomatically, KBD is associated with shortened, enlarged fingers, morning stiffness, arthritic pain, and enlarged joints that are deformed and have inadequate motions at the extremities [3, 4]. The pathology of KBD involves all hyaline cartilage from the endochondral bone, including the articular cartilage and growth plates. In the cartilage tissue of patients with KBD, degenerative changes are evidenced by chondronecrosis at various foci of the deep zone [5]. It differs from osteoarthritis (OA), which involves focal, progressive hyaline articular cartilage loss from the superficial to deep zones.

KBD is an environmental disease and is rarely diagnosed in areas where it is non-endemic [4, 6]. Its etiology has not been clearly established; however, low levels of selenium (Se) contribute to its development. In KBD-endemic areas, Se levels in serum and food are significantly lower than in non-KBD-endemic areas [7–10]. Se supplementation reduces the incidence of KDB in healthy children and improves clinical symptoms in children with the disease [11]. Exposure to the mycotoxin T-2 toxin is also a risk factor [10, 12, 13]. In our previous studies, KBD rat models were established by administering T-2 toxin to rats fed a diet low in Se [14]. This model effectively initiated chondrocyte apoptosis within the articular cartilage deep zone, comparable to KBD [15]. We used the KBD rat model in the present study.

KBD can affect the skeleton, which arises via endochondral bone formation [16]. During endochondral bone formation, progressive changes occur in the cellular features and expression patterns of chondrocytes. In contrast, KBD lesions are accompanied by upregulated chondrocyte differentiation-associated genes, which regulate chondrocyte hypertrophy during endochondral bone formation [17]. Among the pathological features of KBD, abnormal endochondral ossification occurs, involving terminal differentiation of chondrocytes and de-differentiation of KBD chondrocytes. Transforming growth factor (TGF)-β signaling stabilizes the phenotype of pre-hypertrophic chondrocytes and regulates various cellular processes via its type I and II receptors (TGF-βRI and TGF-βRII), which are highly expressed in mineralizing and hypertrophic zones [18]—the target zones of KBD. We hypothesized that TGF-βRI and II are overexpressed in the cartilage of patients with KBD. In the present study, we evaluated TGF-βRI and II expression in KBD donors and KBD rat models.

## Methods

### Tissue specimens from patients

Approval for this study was obtained from the Human and Ethical Committee for Medical Research at Xi’an Jiaotong University (#0075). All child donor samples were collected with the consent of the patients’ guardians. Cartilage specimens were collected from the hand phalanges of eight KBD donors aged 3–12 years and eight control donors aged 3.5–7 years. Control donors were from non-KBD-endemic areas, while the KBD donors were from disease-prone areas. All the donors had died from acute diarrhea, pneumonia, or fatal road traffic accidents. The diagnosis of KBD was based on national guidelines for KDB diagnosis in China (diagnostic code: S/T 207–2010). Diagnoses were performed using right hand radiographic imaging after the cartilage samples were stained using hematoxylin and eosin (H&E). Cartilage sections from the control donors were histologically confirmed using H&E staining. The donor characteristics are shown in Table 1.

**Table 1:** Donors Characteristics

	Number	Male/Female	Age (Mean ± SD)
Control Donors	8	4/4	5.6 ± 3.1
KBD Donors	8	4/4	5.2 ± 1.3

χ_gender_^2^ = 0.000, P_gender_ = 1.000; t = 0.373; P_age_ = 0.714

### Experimental animal samples

**Fig. 1:**
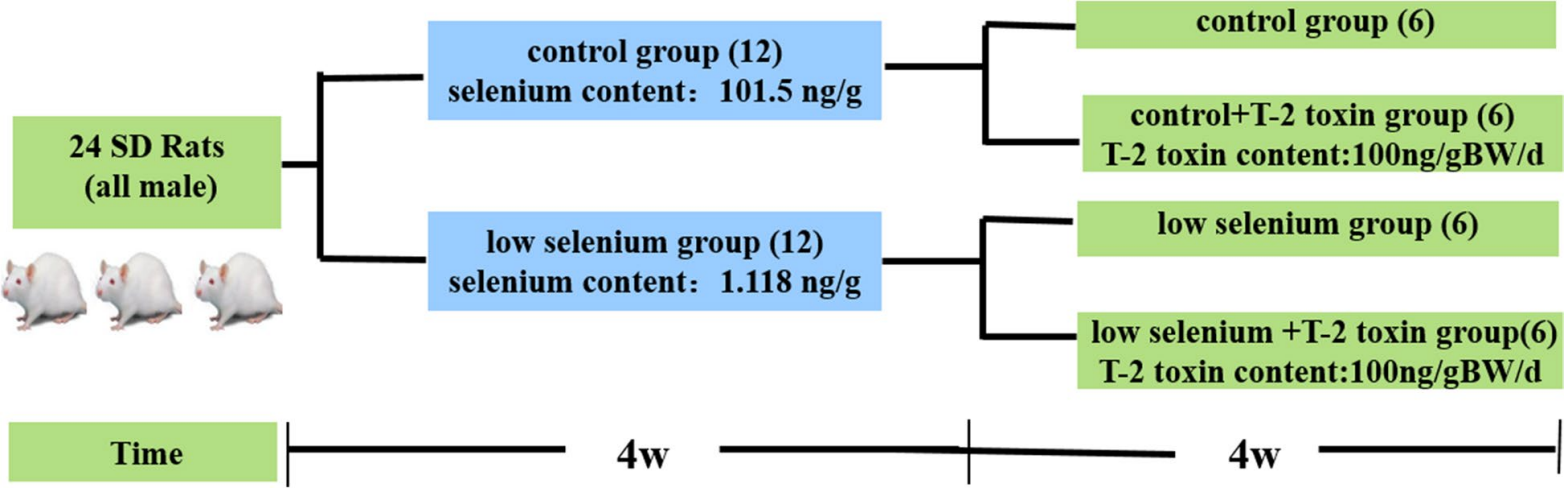
Schematic representation of Experimental animal

Male Sprague–Dawley rats were obtained from the Experimental Animal Center of Xi’an Jiaotong University. Mimicry of human adolescents, who are KDB-susceptible, was established in 1-month-old animals weighing 60–80 g. As previous study [19], rats were randomized into two groups and fed either an Se-deficient diet (Se content: 1.118 ng/g) or a normal diet (Se content: 101.5 ng/g) for 4 weeks. The blood Se levels and serum glutathione peroxidase (GSH-Px) activity levels were then determined to confirm the Se status in these low-Se and normal diet rats. The two groups were then each subdivided into four sub-groups, which were fed either a normal diet (*n* = 6), an Se-deficient diet (*n* = 6), a normal diet supplemented with T-2 toxin at 200 ng/g body weight/day (*n* = 6), or an Se-deficient diet supplemented with T-2 toxin at 200 ng/g body weight/day (*n* = 6). These diets were continued for 4 weeks (Fig. 1). Rat cadavers were obtained after euthanasia by intraperitoneal injection with an overdose of barbiturate (200 mg/kg). The knee joints were processed for histopathological evaluation, and the rats’ costal cartilage was processed for western blot evaluation. The rats’ corpses were collected and processed by the Experimental Animal Center of Xi’an Jiaotong University in accordance with standard principles of animal experimental ethics.

The T-2 toxin was a gift from Prof. Peng Shuangqin and Yang Jinsheng of the Institute of Pharmacology and Toxicology, Academy of Military Medical Sciences. Animal care was undertaken according to the guidelines of the National Institutes of Health publication 85–23 “Guide for Care and Use of Laboratory Animals” (National Research Council, 1996).

### Histology

For 48 h, specimens (human hand phalanges, rat knee) were fixed in neutral buffered formalin (10%); they were then decalcified for 3–4 days using immunocal (Decal; Congers, NY, USA) at room temperature. After dehydration, the samples were embedded in paraffin. Next, 5-μm-thick sections were cut in the sagittal plane. General tissue structures and cell morphologies were evaluated by H&E staining.

For TGF-βR I and II immunohistochemical staining, sample sections were incubated with primary antibodies (Santa Cruz, Dallas, TX, USA) overnight at 4 °C; they were then visualized using alkaline phosphatase-labeled secondary antibodies. Hematoxylin was used to stain the nuclei. Sections were examined by light microscopy and imaged using a digital camera (Canon Corporation, Japan). In each image, positively and negatively stained cells in the three articular cartilage zones were labeled and enumerated using ImageJ software (NIH, USA). Six random fields in each zone were selected and enumerated at 400× magnification. Images were independently analyzed by two observers who were blinded to the study objectives and outcomes. The positive rate of the cells was then calculated using the following equation:

$$The\ percentage\ of\ postive\ cells=\frac{\ positive\ stained\ cells}{\ positive\ stained\ cells+ negative\ stained\ cells}\times 100\%$$

### Western blot

Rat costal cartilage samples were homogenized and extracted using RIPA lysis buffer (Beyotime, Nanjing, China), after which protein levels were determined using a BCA™ protein assay kit (Pierce, Bonn, Germany).

The total protein (30 μg/lane) of each sample was separated using PVDF membranes, which were then incubated with primary antibodies to TGFβRI/II (Santa Cruz, Dallas, TX, USA), and GAPDH (Bioss, Beijing, China) at 37 °C for 30 min and then at 4 °C overnight. Protein levels were standardized against those of GAPDH.

### Statistical analysis

Data are expressed as mean ± standard deviation and were tested for normality and variance. Means were compared using either Student’s t-test or one-way ANOVA followed by Bonferroni’s post hoc. SPSS 18.0 (SPSS Inc., Chicago, IL, USA) was used for statistical analyses. The significance level was set at *p*-values ≤0.05.

## Results

### Histochemical analysis

**Fig. 2:**
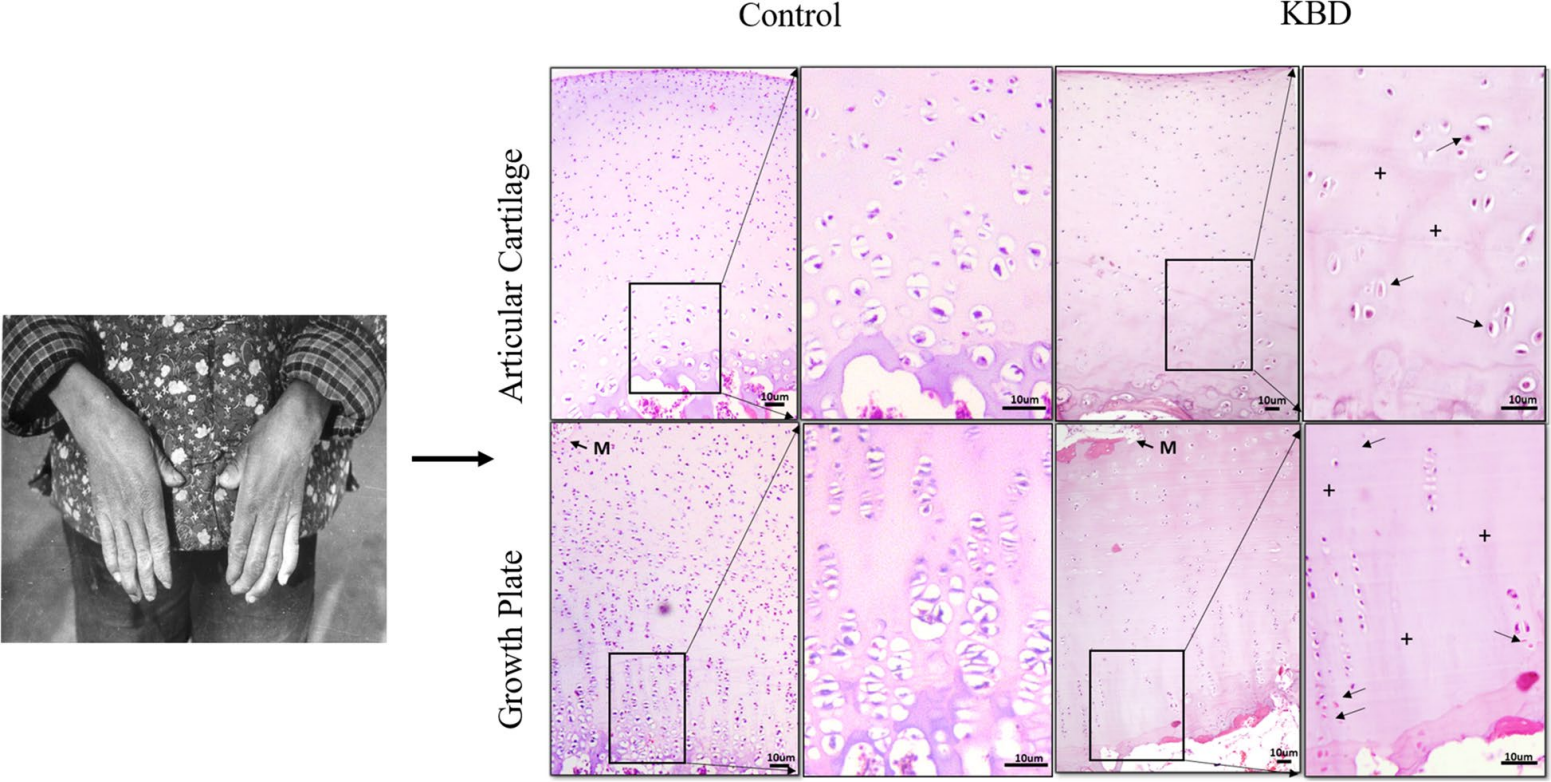
(**A**) A 15-year-old, female patient with KBD, manifested as enlarged phalangeal joints and shortened fingers. (**B**) H&E staining of articular cartilage and growth plate cartilage from the phalanges. “Red ghost” outlines of the chondrocytes can be seen in the deep zone (black arrow). No chondrocytes are present in the deep zone of cartilage (+)

Figure 2 shows H&E stained images of cartilage from donors with and without KBD. Necrotic fields were characteristically observed at the zones of the maturing cartilage and extended to the transition region between the hypertrophic and proliferative zones of growth plate cartilage, or to the middle and deep zones of the articular cartilages. This was not observed in control cartilage (Fig. 2). In donors with KBD, chondral necrosis was established based on cartilage cell disappearance, whereby only red outlines of chondrocytes remained, accompanied by alkalinity loss in the matrix ground substance, which exhibited a lighter blue color upon H&E staining.

### Histomorphological analysis of TGF-βRI and TGF-βRII in the cartilage of patients with KBD

**Fig. 3:**
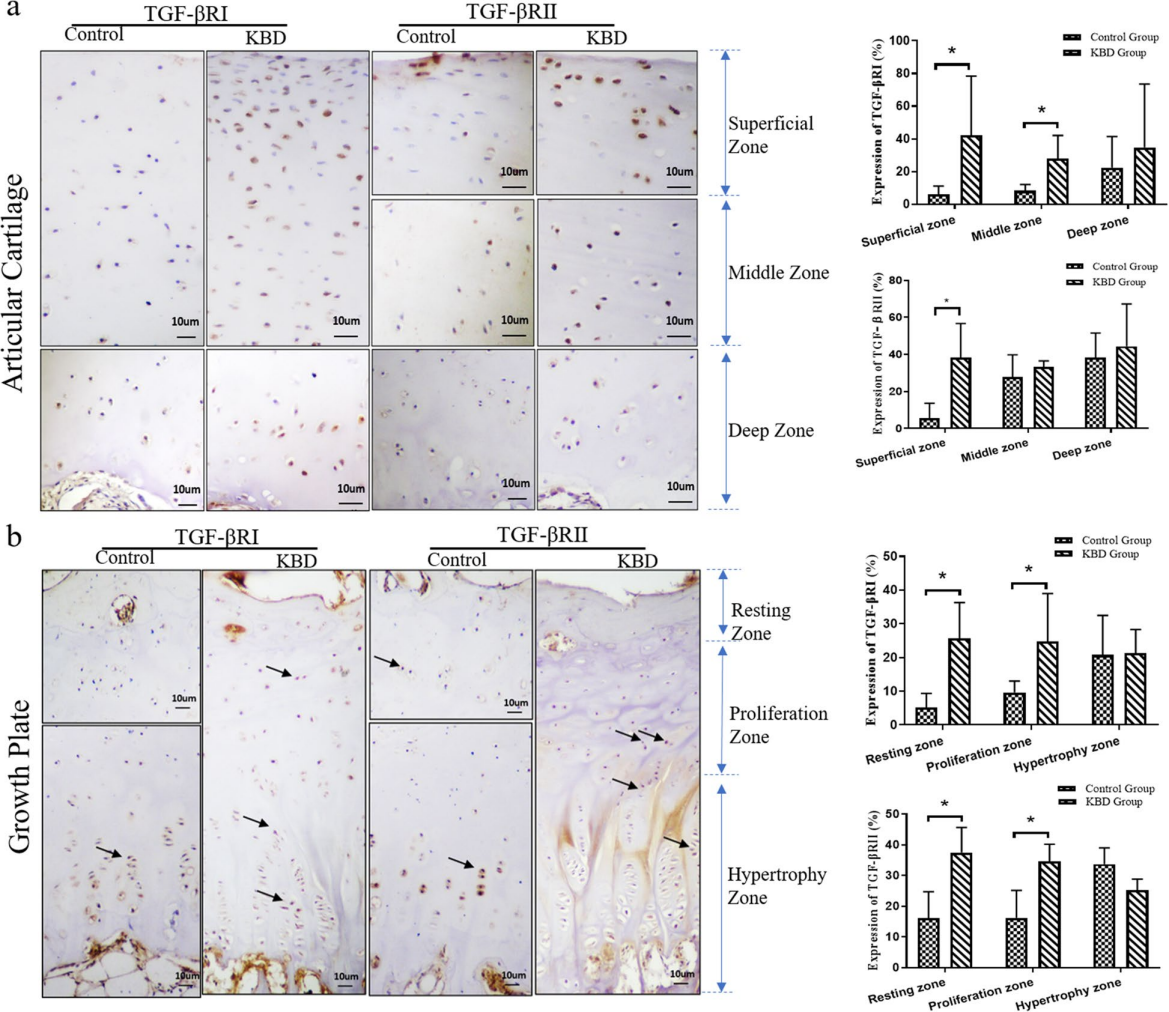
Immunostaining of TGFβRI and TGFβRII in control and KBD cartilage (positive staining is brown, indicated with an arrow). (**A**) Staining of TGFβRI and TGFβRII in articular cartilage. (**B**) Staining of TGFβRI and TGFβRII in growth plate cartilage (*n* = 6; *p < 0.05)

An antibody recognizing TGF-βRI/II was used to immunolocalize these proteins in the phalange cartilage of donors with KBD. Both TGFβRI and TGFβRII were localized in the membrane and adjacent cytoplasm. Positive staining of chondrocytes for TGFβRI/II was weak in the cartilage depth from control donors (Fig. 3a, b). However, TGF-βRI/II expression was detected throughout the KBD cartilage samples, with strong staining in the superficial and middle articular cartilages (Fig. 3a) and in the resting and proliferation zones of the growth plate cartilage (Fig. 3b).

### Histomorphological analysis of TGF-βRI and TGF-βRII in the cartilage of the KBD rat model

**Fig. 4:**
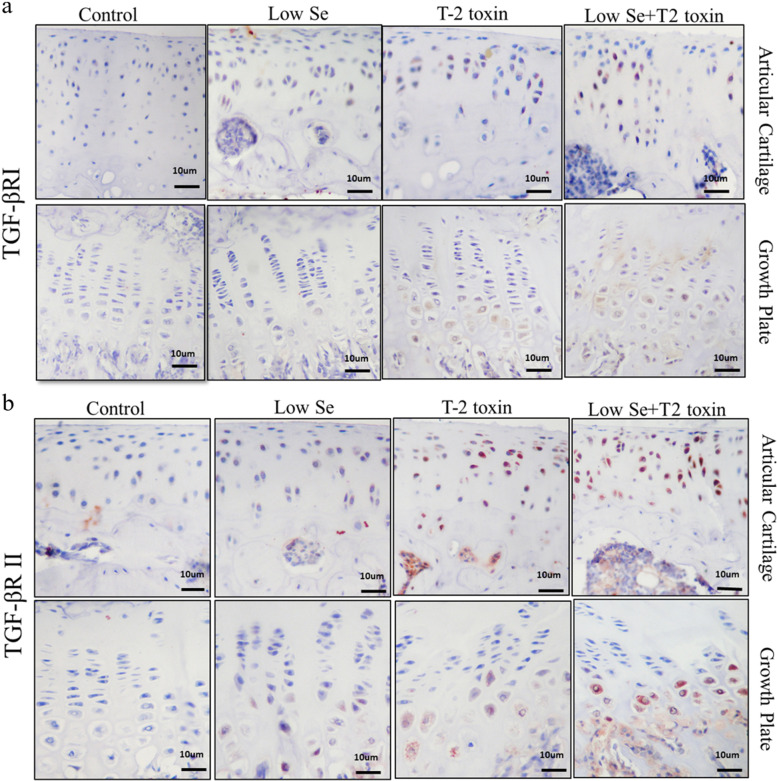
Representative immunostaining showing TGFβRI and TGFβRII expression in the knee joints of rats assessed using immunohistochemistry (positive staining is red, indicated with an arrow). (**A**) TGFβRI staining in articular and growth plate cartilage. (**B**) TGFβRII staining in articular and growth plate cartilage

To provide more insight into the correlation between TGF-βRI/II and KBD, we detected TGF-βRI/II localization and expression in the knee articular and growth plate cartilage of KBD rats. In humans, both TGFβRI and TGFβRII were localized in the membranes and adjacent cytoplasms (Fig. 4a, b). Positive staining of chondrocytes for TGFβRI/II was weak in the cartilage of rats fed normal or Se-deficient diets (Fig. 4a, b). Strong TGF-βRI/II staining was detected in the cartilage of rats treated with T-2 toxin alone or with T-2 toxin and Se-deficient feed, particularly in the middle and deep zones of articular cartilage and in the proliferation zones of growth plate cartilage (Fig. 4a, b).

### Western blotting of TGF-βRI and TGF-βRII in cartilage from KBD rat models

**Fig. 5:**
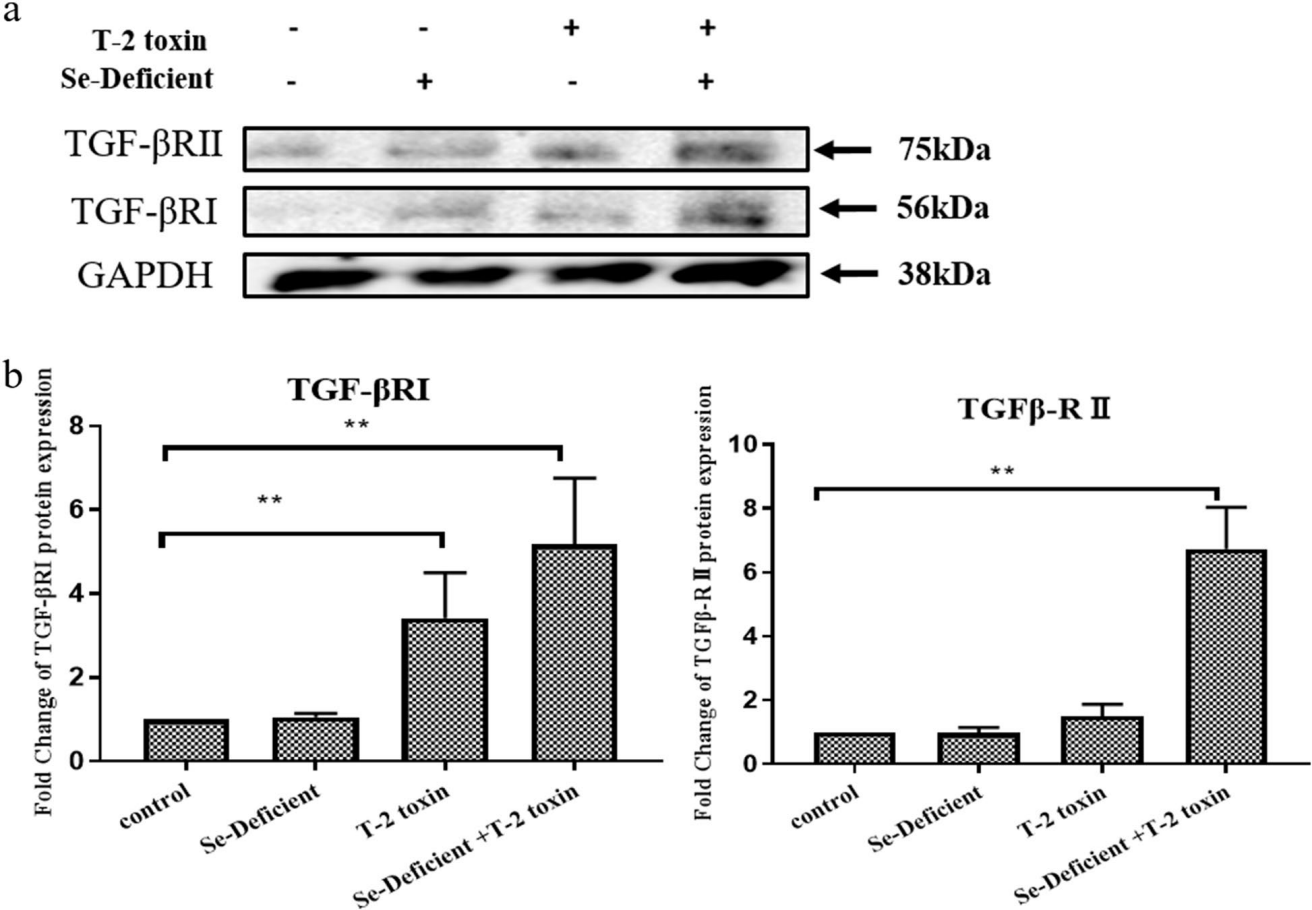
TGF-βRI and TGF-βRII protein expression levels in rat cartilage samples. (**A**) Representative images showing TGF-βRI and TGF-βRII protein expression in various samples. (**B**) Quantitative data of TGF-βRI and TGF-βRII protein expression levels in various samples. Results were normalized to GAPDH levels (*n* = 6; **p < 0.01). The gels were cropped from different parts of the same gel and transferred to membranes with different transfer times (semi-dry transfer system: GAPDH, 25 min, 15 V; TGFβRI/II, 40 min, 15 V)

We used western blot analysis to quantify TGF-βRI and II protein expression levels in the KBD rat model (Fig. 5). TGF-βRI and II protein levels were markedly higher in rats fed a T-2 toxin diet and in those fed a T-2 toxin-supplemented, Se-deficient diet (Fig. 5a) than in those fed a normal diet or an Se-deficient diet. TGF-βRI protein expression increased 3-fold in rats fed T-2 toxin diets and 5-fold in rats fed T-2 toxin and Se-deficient diets (Fig. 5b). TGF-βRII protein levels were approximately 6-fold higher in rats treated with a T-2 toxin plus Se-deficient diet than in those fed a normal diet (Fig. 5b).

## Discussion

The pathology of KBD involves all hyaline cartilages from endochondral bone, including the articular cartilage and growth plates. As shown in Fig. 2, degenerative changes were observed in KBD cartilages. They were characterized by chondronecrosis in multiple foci of cartilage deep zones [5]. In this way, KBD differs from OA, which involves focal and progressive hyaline articular cartilage loss from the superficial to the deep zones. Pathological changes in the deep zone of cartilage involve extracellular matrix (ECM) destruction and resorption. The ECM is mainly composed of proteoglycan aggrecan, type X collagen, and other matrix molecules [20]. Excess type X collagen cleavage in KBD is associated with upregulated collagenase synthesis and activities, especially matrix metalloproteinase (MMP)-13 [21, 22].

Apart from the cranial vault bones, parts of the jaw, and the medial part of the clavicle, KBD can occur at any point in the skeleton. Its etiology is correlated with endochondral bone formation [16], during which there is progressive change in the cellular characteristics and expression patterns of chondrocytes. In the present study, markedly increased expression of TGF-βRI and TGF-βRII was observed in KBD samples (Fig. 3), as well as in pre-clinical models of the disease (Figs. 4 and 5). Cartilage erosion in maturing and hypertrophic cartilage zones is a fundamental pathological feature of KBD. As shown in Fig. 4, strong staining of TGF-βRI and TGF-βRII was observed in maturing and hypertrophic cartilage zones in pre-clinical models of this disease. On the other hand, the percentage of positive staining for TGF-βRI and TGF-βRII was significantly higher in the superficial and middle articular cartilages, as well as in the resting and proliferation zones of growth plate cartilage in human samples (Fig. 3). Guo et al. demonstrated that hand lesions are associated with upregulated chondrocyte differentiation-associated genes, which are regulate chondrocyte hypertrophy during endochondral bone formation [17]. These genes TGF-β, whose signaling may therefore be involved in the pathophysiology of KBD.

TGF-β signaling regulates cell differentiation, chemotaxis, proliferation, and ECM production through TGF-βRI and TGF-βRII. TGF-β signaling is induced when ligands bind to the cell surface receptors TGF-βRI/II, which are highly expressed in the mineralizing and hypertrophic zones [18]. TGF-βRII is the only TGF-β receptor that binds all TGF-β isoforms and elicits functional signaling. TGF-β isolation from culture media is essential for stem cell differentiation into hypertrophic chondrocytes [23, 24]. In primary mouse limb bud mesenchymal cell cultures, TGF-β inhibits the expression of the terminal differentiation marker type X collagen [25]. In addition, mice with homozygous disruption of Smad3 exhibited elevated hypertrophic chondrocyte counts [26]. Therefore, TGF-βRI and TGF-βRII overexpression in KBD may prevent hypertrophy of chondrocytes. In an in vivo study [27], MMP13, which is an indicator of collagen expression, was correlated with elevated TGF-β1 expression through the SMAD-independent TGF-β pathway in OA-affected cartilage. Therefore, over-activation of TGF-β signaling may lead to cartilage matrix destruction and abnormal terminal differentiation of chondrocytes.

A low-Se diet and exposure to T-2 toxin are etiological factors associated with KBD. In the present study, TGF-βRI and TGF-βRII expression were sharply increased in the low-Se diet plus T-2 toxin group (Figs. 4 and 5). Recent reports [27, 28] have shown that TGF-β signaling mediates T-2 toxin-induced decreases in type II collagen in cultured rat chondrocytes, and that Se attenuates MMP-2 overexpression by regulating the TGF-β1/Smad signaling pathway. Type II collagen is under-expressed in individuals with KBD, whereas the opposite is true for MMPs. Thus, low-Se plus T-2 toxin may lead to cartilage degeneration through overexpression of the TGF-βR signal, contributing to the development of KBD.

The present study had some important limitations. A relatively small number of patients were included in the study groups. KBD often occurs in children aged 3–13 years and damages cartilage; therefore, it is difficult to obtain fresh samples to extract protein and RNA. We found a relationship between TGF-βRI and II and KBD; however, the underlying mechanism(s) that cause the presence and buildup of these molecules remain unclear.

In conclusion, the TGF-βR signal is over-activated in KBD cartilage. The present study provided new insights into the pathways that contribute to KBD development. In the future, we will continue to focus on this aspect. Since the TGF-βR signal is over-activated in KBD cartilage, we will block the TGF-βR signal in vivo and in vitro to detect changes in cartilage matrix destruction and abnormal terminal differentiation of chondrocytes. In this way, we will determine the role of the TGF-βR signaling pathway in patients with KBD. Finally, we aimed to identify potential therapeutic targets for KBD.

## Supplementary Information

**Additional file 1.**

## Data Availability

The datasets generated and/or analysed during the current study are not publicly available due to limitations of ethical approval involving the patient data and anonymity but are available from the corresponding author on reasonable request.

## Abbreviations

KBD: Kashin-Beck disease
TGF-βR: Transforming growth factor-β receptor
H&E staining: Hematoxylin-eosin staining
OA: Osteoarthritis
FGF: Fibroblast growth factor
Se: Selenium
ECM: Extracellular matrix
MMP: Matrix metalloproteinase
CRR: Chamber replacement rate
