# Supplementary material for: Upregulated expression of transforming growth factor-β receptor I/II in an endemic Osteoarthropathy in China
Source: BMC Musculoskelet Disord. 2021 Dec 20;22:1051. doi: 10.1186/s12891-021-04939-6 (PMC8690967; doi:10.1186/s12891-021-04939-6)

**Upregulated Expression of Transforming Growth Factor-β Receptor I/II**

**in** **an Endemic Osteoarthropathy in China**

Ying. Zhang^1,2^, Yudong. Mu^3^, Ying. He^1^, Zhengzheng. Li^1^, Yinan Liu^1^, Meng. Zhang^1^, Hui. Wang^1^, Yiping. Feng^1^, Qian. Fang^1^, Tianyou. Ma^1^, Xianghua. Deng^4^, Jinghong. Chen^1*^

1. School of Public Health, Health Science Center of Xi'an Jiaotong University, and Key Laboratory of Trace Elements and Endemic Diseases of National Health and Family Planning Commission of the People's Republic of China, and Collaborative Innovation Center of Endemic Diseases and Health Promotion in Silk Road Region, Xi’an, Shaanxi, 710061, P.R. China.
2. School of Nursing, Health Science Center, Xi’an Jiaotong University, Xi’an, Shaanxi, 710061, P.R. China
3. Department of Clinical Laboratory, Tumor Hospital of Shaanxi Province, Affiliated to the Medical Collage of Xi'an Jiaotong University, Xi’an, Shaanxi, 710061, P.R. China.
4. Research Division, HSS, Research Institute, Hospital for Special Surgery, and Weill Cornell Medical College, 535 East 70th Street, New York, New York 10021, USA

*Corresponding Authors: Dr. Jinghong Chen

The Institute of Endemic Disease, Medical School, Xi’an Jiaotong University, Xi’an, Shaanxi, 710061, P.R. China.

Email: [jixiang46@163.com](mailto:jixiang46@163.com)

Tel: +86-029-82655195


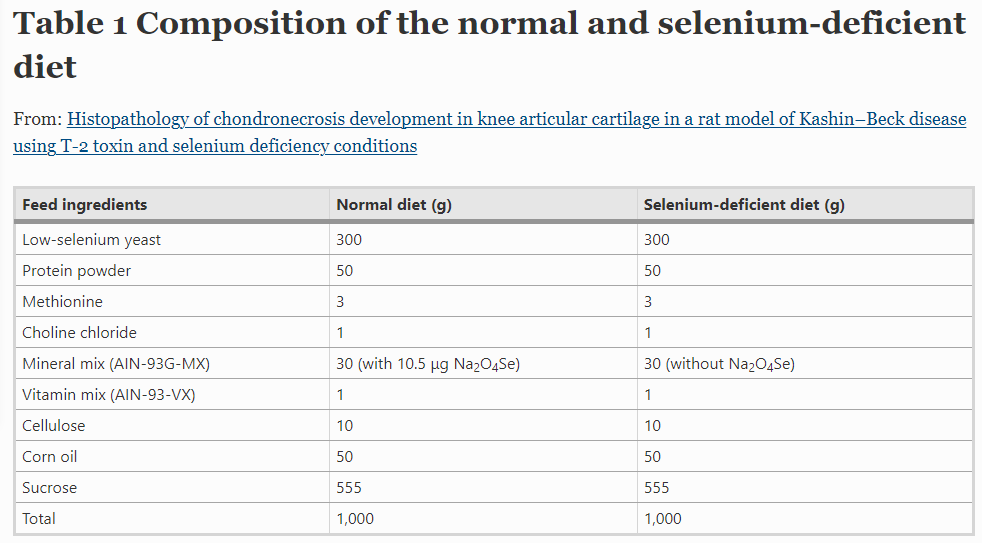


**Composition of the normal and selenium-deficient diet**

Guan F, Li S, Wang ZL, Yang H, Xue S, Wang W, Song D, Zhou X, Zhou W, Chen JH *et al*: **Histopathology of chondronecrosis development in knee articular cartilage in a rat model of Kashin-Beck disease using T-2 toxin and selenium deficiency conditions**. *Rheumatol Int* 2013, **33**(1):157-166.

**Full-length gel (staining with coomassie brilliant blue)**


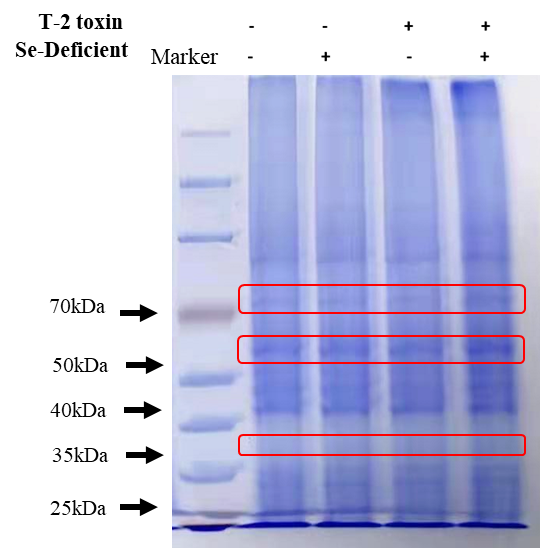


**Full-length membrane**


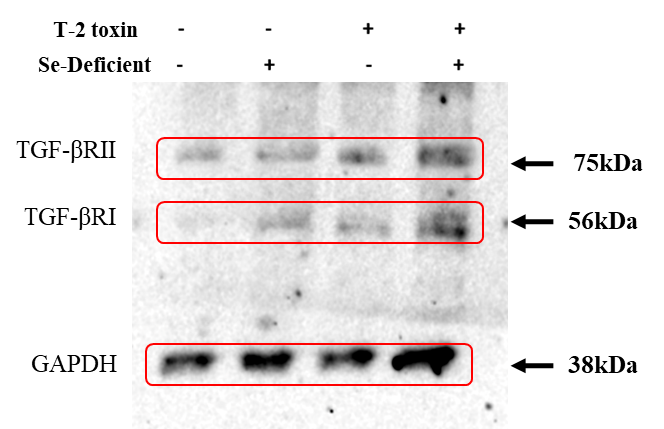

Supplement: Supplementary file 1 — Additional file 1. [file 12891_2021_4939_MOESM1_ESM.docx]
